# Non-varicose gastrointestinal bleeding: an overlooked cause of bleeding in porto-sinusoidal vascular liver disorder

**DOI:** 10.1093/gastro/goaf101

**Published:** 2025-11-22

**Authors:** Xiaoru Sun, Liqin Shi, Rongtao Lai, Xiaojin Wang, Muyun Liu

**Affiliations:** Department of Infectious Disease, NO. 905 Hospital of PLA Navy Affiliated to Naval Medical University, Shanghai, P. R. China; Department of Gastroenterology, National Clinical Research Center for Digestive Diseases, Changhai Hospital, National Key Laboratory of Immunity and Inflammation, Naval Medical University, Shanghai, P. R. China; Department of Infectious Disease, NO. 905 Hospital of PLA Navy Affiliated to Naval Medical University, Shanghai, P. R. China; Department of Infectious Diseases, Ruijin Hospital, Shanghai Jiaotong University School of Medicine, Shanghai, P. R. China; Editorial Department of Journal, Publishing House of Chinese Hepatology, Shanghai,P. R. China; Department of Infectious Disease, NO. 905 Hospital of PLA Navy Affiliated to Naval Medical University, Shanghai, P. R. China; Department of Gastroenterology, National Clinical Research Center for Digestive Diseases, Changhai Hospital, National Key Laboratory of Immunity and Inflammation, Naval Medical University, Shanghai, P. R. China; Department of Gastroenterology, NO. 905 Hospital of PLA Navy Affiliated to Naval Medical University, Shanghai, P. R. China

## Introduction

Porto-sinusoidal vascular liver disorder (PSVD), including idiopathic noncirrhotic portal hypertension (PH), is a group of rare vascular liver diseases affecting the portal veins or hepatic sinusoids [1, 2]. According to statistics, approximately half of patients with PSVD have coexisting PH [3, 4]. Current studies have shown that PSVD may be linked to genetics, chronic infection, immune disorders, hypercoagulability, and exposure to drugs and toxicants [1]. Common clinical manifestations in these patients include gastroesophageal or anorectal varices and splenomegaly, with liver function remaining largely intact [5]. Gastrointestinal bleeding in patients with PSVD is primarily caused by the rupture of varices resulting from PH. Such bleeding typically occurs in the esophagus and stomach, and rarely in the small intestine. At present, insufficient attention is being paid to non-variceal gastrointestinal bleeding in atypical sites in PSVD cases worldwide. Here, we reported a case of PSVD combined with portal hypertensive enteropathy leading to recurrent gastrointestinal bleeding. This case has an innovative significance in supplementing the disease spectrum of PSVD.

## Case report

**Figure 1.**
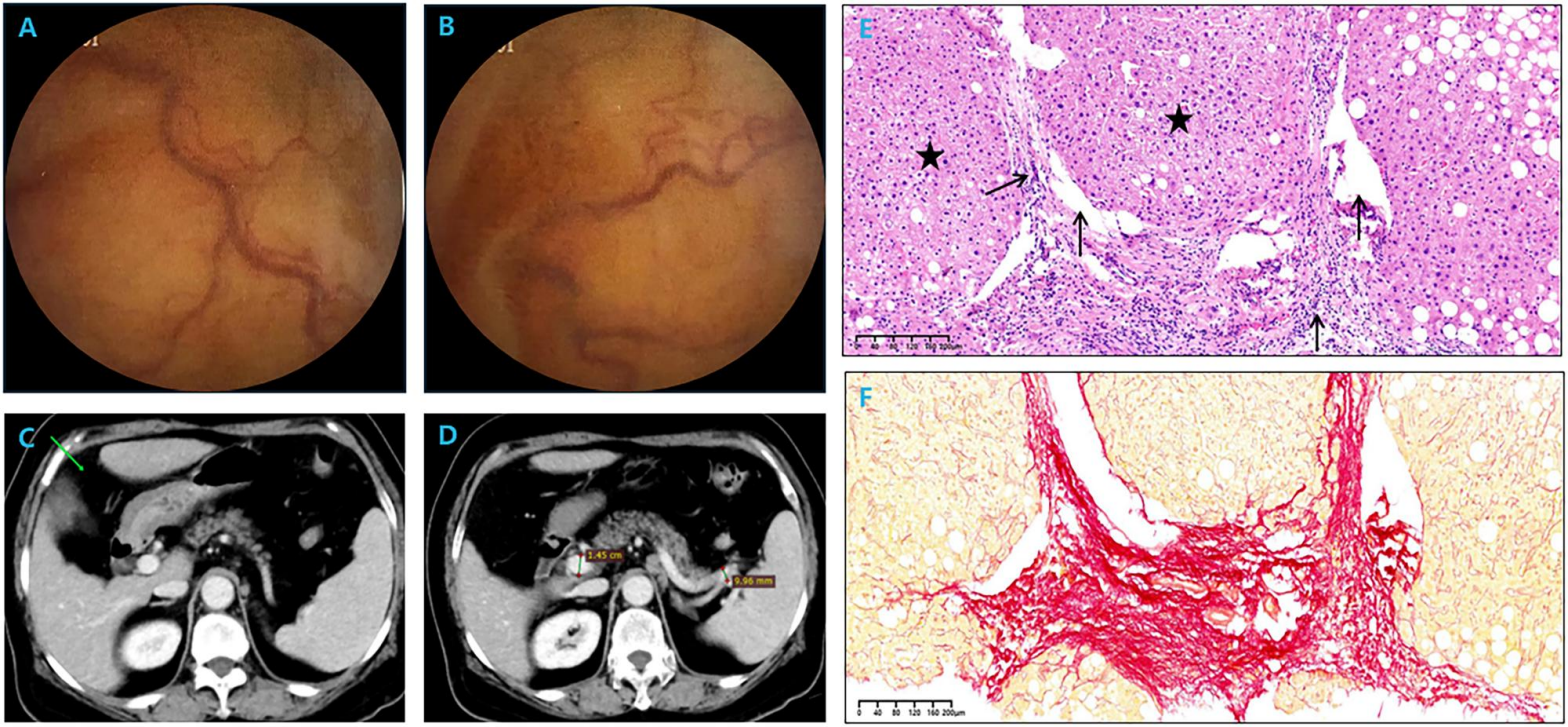
Clinical data of the case. (A, B) Capsule endoscopy revealing scattered erythema and slightly twisted and dilated blood vessels in the small intestine. (C) Hepatic CT angiography showing slightly wider hepatic fissure. (D) Hepatic CT angiography showing mild splenomegaly and mild dilatation of the portal vein and splenic vein. (E) HE staining showing moderate inflammation in the portal area, portal phlebitis and periphlebitis with periportal fibrosis, fibrous septa formation, portal vein stenosis in a fragmented manner, partial portal vein herniation into adjacent hepatic parenchyma (arrows), and nodular regeneration of hepatocytes (asterisks) (×4). (F) Sirius red staining showing continuous fibrous septa in the portal vein area (×10).

A 69-year-old woman presented with chronic black stools and declining hemoglobin levels over 4 years, without a history of skin or mucosal bleeding, radiation or toxin exposure, or alcohol abuse. Gastroscopy and colonoscopy showed no signs of abnormal bleeding. One year prior, capsule endoscopy had revealed scattered erythema and slightly twisted and dilated blood vessels in the small intestine (Figure 1A and B), potentially linked to her black stool but with an undetermined etiology. Therefore, only medical symptomatic treatment for gastrointestinal bleeding could be provided to the patient and the patient still had recurrent melena after discharge. Six months ago, the patient underwent laparoscopic cholecystectomy for gallstones, during which small nodules on the surface of the liver and splenomegaly were observed. Liver transient elastography showed a significantly elevated liver stiffness of 15.8 kPa. Combined with capsule endoscopic findings, it was considered to be portal hypertensive enteropathy caused by PH in liver cirrhosis. However, serological tests for hepatitis viruses, Epstein–Barr virus, cytomegalovirus, human immunodeficiency virus, and syphilis were negative. Autoantibodies were negative and serum immunoglobulin levels were within normal limits. Whole-exome gene screening did not detect any variations related to the patient’s clinical symptoms and copper metabolism indicators were also normal. An echocardiogram showed no abnormalities. Hematologic evaluation showed only mild elevations of aspartate aminotransferase and gamma-glutamyl transferase. Hepatic CT angiography did not show vascular obstruction or emboli, but revealed slightly wider hepatic fissure, slightly thickened hepatic echo, mild splenomegaly, and mild dilatation of the portal vein and splenic vein (Figure 1C and D). To further clarify the nature of the liver lesions, we performed a liver biopsy.

The liver biopsy demonstrated a disordered lobular architecture, portal stenosis, nodular regenerative hyperplasia, incomplete septal fibrosis, and herniation of the portal vein into the hepatic parenchyma (Figure 1E and F). These findings are consistent with the pathological manifestations of PSVD. Although the patient did not exhibit symptoms of melena during this visit, we still administered propranolol and iron supplementation. Since discharge, the patient has not experienced melena again over the course of 9 months, and the red blood cell count and hemoglobin levels have returned to normal.

## Discussion

PSVD is defined as vascular liver disease characterized by the absence of cirrhosis on liver biopsy and the presence of histological lesions suggestive of this disease (such as obliterative portal venopathy, nodular regenerative hyperplasia, incomplete septal fibrosis), with or without the presence of PH [2]. Histopathology plays a crucial role in diagnosing PSVD and distinguishes PSVD from cirrhosis and other portal hypertensive liver diseases [2].

Studies indicate that 44%–59.3% of PSVD patients have PH [3, 4]. The abnormal increase in portal pressure and the formation of collateral circulation caused by PH are the main causes of gastrointestinal bleeding in PSVD patients [3, 4]. Recent large observational studies indicate that, in PSVD, gastrointestinal bleeding most often originates from the esophagus and stomach. Bleeding from ectopic varices or from portal hypertensive mucosal disease is extremely rare and seldom documented. Although several studies have suggested that mucosal lesions may cause chronic occult gastrointestinal bleeding in PSVD patients, no relevant cases have been reported prior to this one [3–5]. This case, therefore, provides a documented example of this rare complication.

No specific treatment protocol exists for PSVD patients with PH. Physicians primarily manage PH. Nonselective beta-blockers are often effective for mild to moderate cases. Severe complications, such as recurrent esophageal–gastric variceal bleeding or refractory ascites, often require a transjugular intrahepatic portosystemic shunt. Physicians may recommend splenic embolization or splenectomy for refractory hypersplenism [6, 7]. This patient received oral propranolol and iron supplementation for 1 year without recurrent gastrointestinal bleeding and the hemoglobin levels returned to normal. This outcome demonstrates that controlling PH early greatly improves prognosis.

In summary, recurrent small bowel bleeding in PSVD due to portal hypertensive enteropathy is rarely reported. This report documents a pathologically confirmed case of such bleeding that achieved long-term remission. Early recognition, careful evaluation, and pressure-lowering treatment are essential for better patient outcomes.

## Authors’ contributions

X.S., M.L., and L.S. conceived the article, participated in its design and coordination, and drafted the manuscript. R.L. and X.W. collected clinical data and performed the literature review. M.L. and R.L. revised the important intellectual content of the manuscript. All authors read and approved the final manuscript.
